# A novel heterozygous mutation of the *NPHS1* gene in a Chinese child with congenital nephrotic syndrome: A case report

**DOI:** 10.1097/MD.0000000000032970

**Published:** 2023-02-17

**Authors:** Dan Xie, Jiangfen Wu, Wenyi Zhang, Tingting Jin, Peng Wu, Banquan An, Shengwen Huang

**Affiliations:** a Medical College, Guizhou University, Guiyang, China; b Department of Laboratory Medicine, Guizhou Provincial People’s Hospital, Guiyang, Guizhou, China; c Prenatal Diagnostic Center, Guizhou Provincial People’s Hospital, Guiyang, Guizhou, China; d Discipline Inspection and Supervision Office, Guizhou Provincial People’s Hospital, Guiyang, Guizhou, China.

**Keywords:** congenital nephrotic syndrome, mutation, *NPHS1*, whole exome sequencing

## Abstract

Congenital nephrotic syndrome (CNS) is a heterogeneous disorder in which massive proteinuria, hypoproteinemia, and hyperlipidemia and marked edema are the main manifestations before 3 months-of-age. Here, we present a case involving the genetic diagnosis of a child with CNS.

**Patient concerns:**

A 31-day-old male infant with diarrhea for 25 days and generalized edema for more than 10 days. There was no family history of kidney disease. On proband whole exome sequencing, a compound heterozygous mutation of the *NPHS1* gene was identified, including a novel in-frame mutation in exon 14 (c.1864_1866dupACC p. T622dup) and a missense mutation in exon 8 (c.928G>A p. D310N).

**Diagnoses:**

Based on the clinical and genetic findings, this patient was finally diagnosed with CNS.

**Interventions:**

The main treatment options for the patient were 2-fold: anti-infective treatment and symptomatic treatment.

**Outcomes:**

The patient died in follow-up 2 months later; the specific reason for death was unclear.

**Lessons:**

Whole exome sequencing and Sanger sequencing confirmed that the infant had CNS. Our study identified a novel mutation in an infant, thus expanding the gene-mutation spectrum of the *NPHS1* gene, thus providing an efficient prenatal screening strategy and early genetic counseling.

## 1. Introduction

Congenital nephrotic syndrome (CNS) is a rare disease that is characterized by the development of a large placenta within the first 3 months of gestation and early onset (infancy, neonatal or antenatal).^[1,2]^ The estimated incidence of CNS is 1 to 3 per 100,000 children worldwide.^[3]^ Notably, CNS can be differentiated from infantile nephrotic syndrome, which appears later during the first 1 to 2 years of life and mostly has a more favorable prognosis.^[4]^ The most common type of (CNS is the Finnish type [CNF], MIM: 256300); this is a recessively inherited disorder that was first described in highly inbred Finnish communities.^[5,6]^

Herein, we used whole exome sequencing (WES) to identify and characterize 2 heterozygous mutations in exon 8 and exon 14 of the *NPHS1* gene (NM_004646: c.928G>A, c.1864_1866dupACC) from a Chinese infant with CNS. The second mutation is an in-frame mutation and leads to the insertion of an amino acid (p. T622dup). This mutation site is the first reported to be reported and expands the mutational spectrum of the *NPHS1* gene.

## 2. Case report

The patient was a boy aged 31 days. He was the first child of a nonconsanguineous, 28-year-old, G3P1 mother and a 32-year-old father. The mother had a history of upper respiratory tract infection in early pregnancy and was not treated with medication. Aside from that, her plasma alpha-fetoprotein (AFP) level was significantly elevated during pregnancy, although the specific levels were not recorded.

**Figure 1.**
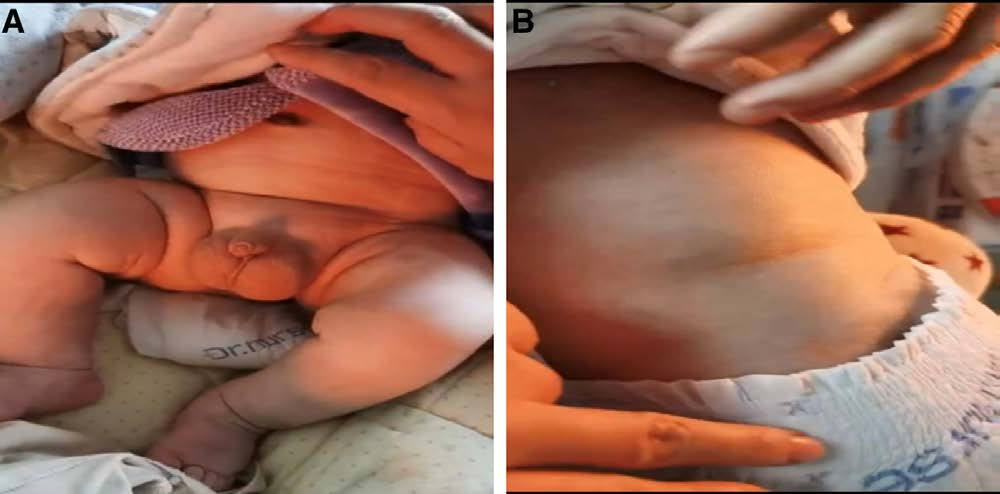
Clinical images of the proband. (A) Severe edema of the lower limbs and (B) abdominal distention.

The patient was admitted to the hospital with a history of diarrhea for 25 days and generalized edema for more than 10 days. He was a full-term normal delivery baby with a birth weight of 3415 g. At delivery, the placenta was large, and the umbilical cord was thick. However, the weight of his placenta was not recorded. A physical examination on admission showed a temperature of 36.6°C, a respiratory rate of 30 breaths/minute, and a pulse rate of 160 beats/minute. He also exhibited facial and bilateral eyelid edema and mild pitting edema in both lower limbs (Fig. 1A, B).

The results of a laboratory examination are shown in Table 1. There was evidence of hyperkalemia, with a blood potassium level of 5.88 mmol/L (normal levels, 3.5–5.3 mmol/L), hyperlactatemia, and with a lactic acid level of 4.28 mmol/L (normal levels, 0.5–2.2 mmol/L). Secondary immune deficiency was also observed (IgG < 1.0900 g/L [normal levels, 2.03–9.34 g/L]). Tests for toxoplasma, rubella, and cytomegalovirus and herpes simplex (TORCH) infections were negative. Renal vascular color ultrasound revealed an increased resistance index in both renal arteries and echocardiography revealed a patent foramen ovale with left-to-right separation at the level of the foramen ovale.

**Table 1:** Laboratory results.

Parameter	Presentation	Reference ranges
Urinary protein	Positive (3+)	Negative
Vitamin C	Positive (2+)	Negative
Urinary occult blood	Weak positive (+−)	Negative
Microalbumin (mALB)	2341.08	0–19 mg/L
Urine transferrin (UTFN)	192.440	0–2 mg/L
Albumin/creatinine ratio (ACR)	4169.3	0–3 mg/mmol
White blood cell (WBC)	11.15 × 10^9	6–8 × 10^9/L
Red blood cell (RBC)	4.43 × 10^12	4.5–6.5 × 10^12/L
Hemoglobin (HGB)	161.0	95–145 g/L
Platelet (PLT)	377 × 10^9/	125–350 × 10^9/L
Total protein (TP)	308	44–76 g/L
Albumin (ALB)	14.7	38–54 g/L
Globulin (GLB)	16.1	20–40 g/L
Aspartate aminotransferase (AST)	35	13–35 U/L
Alanine aminotransferase (ALT)	13	7–40 U/L

The main treatment options for the patient were 2-fold. anti-infective treatment was performed with penicillin sodium. Symptomatic treatment included human albumin to compensate for albumin, montmorillonite suspension for antidiarrheal treatment, bifidobacterium triple combination bacteria to regulate intestinal bacteria, and vitamin K1 to prevent late-onset vitamin K1 deficiency. After 2 weeks of treatment, there were still edema and hyperkalemia; treatment needed to be continued but the family requested discharge. Unfortunately, the patient died in follow-up 2 months later; the specific reason for death was unclear. Congenital nephrotic syndrome was considered based on the child physical and laboratory findings. There was no family history of kidney disease.

### 2.1. Genetic analysis

Genomic DNA was extracted from samples of whole blood taken from the patient and his parents using the QIAamp DNA Mini Kit (Qiagen, Shanghai, China) according to the manufacturer instructions. The DNA was then quantified with a Nanodrop 2000 (Thermal Fisher Scientific, DE). Genomic DNA (1–3 μg) was fragmented to an average size of 150 bp using an S220 Focused-ultrasonicator (Covaris, Massachusetts). A DNA Sample Prep Reagent Set (MyGenostics, Beijing, China) was then used to prepare standard libraries, including end repair, adapter ligation, PCR amplification, and which were then sequenced by DNBSEQ (DNBSEQ-T7).

**Figure 2.**
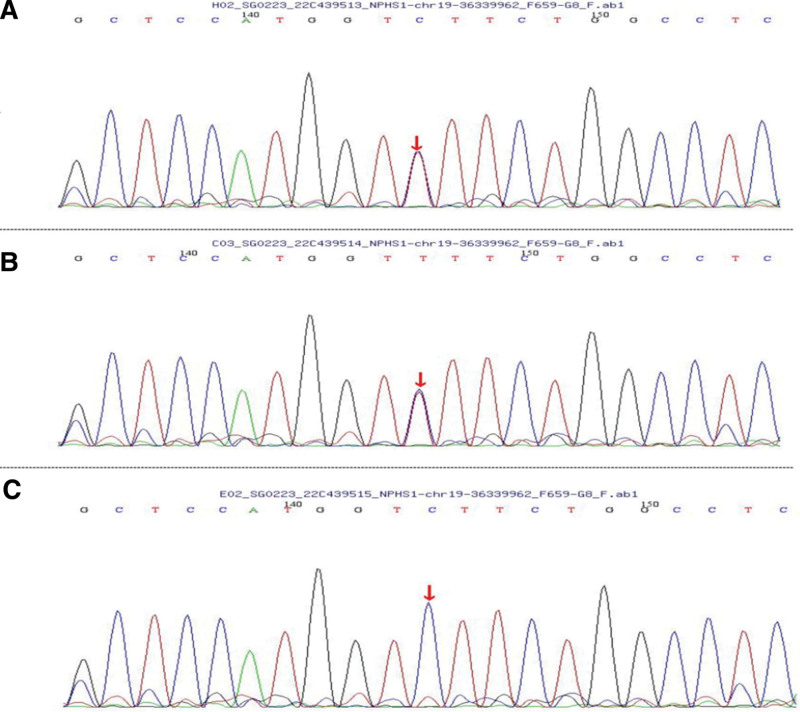
Chromatogram showing the c.928G>A mutation, obtained by Sanger sequencing, in the proband and his parents. The red triangle indicates the 928 site, (A) the proband, (B) the proband father, and (C) the proband mother.

**Figure 3.**
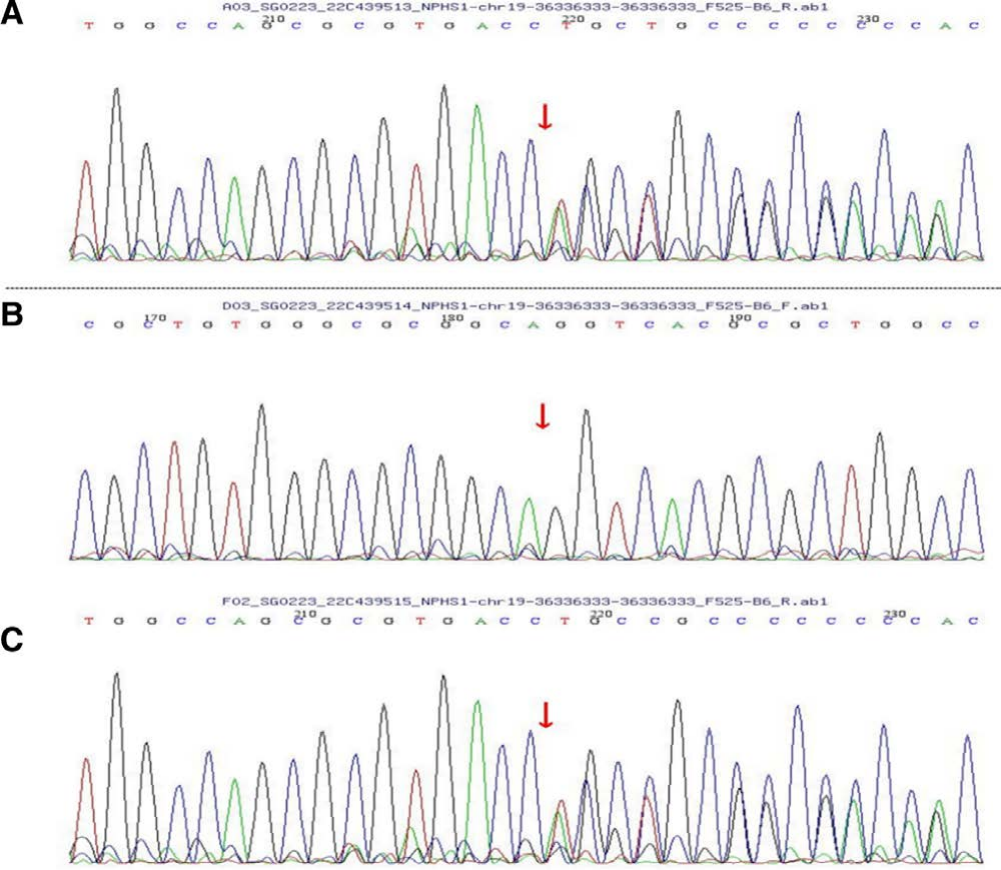
Chromatograms of the c.1864_1866dupACC mutation, obtained by Sanger sequencing, in the proband and his parents. (A) The proband, (B) the proband father, and (C) the proband mother.

The amplified DNA was captured by a GenCap Whole-exome System Capture Kit (MyGenostics Inc, Beijing, China). Biotinylated 100 bp capture probes were designed to tile along the coding exons plus 50 bp flanking regions of all genes. The capture experiment was conducted according to the manufacturer protocol. The enrichment libraries were sequenced on a (DNBSEQ-T7) sequencer for the paired reading of 150 bp fragments. WES identified 2 heterozygous mutations (c.928G>A and c.1864_1866dupACC) in NPHS1. The c.928G>A mutation has been reported previously and can cause an amino acid substitution (D310N) associated with CNS.^[2]^ The novel in-frame mutation, c. 1864_1866dupACC, and within exon 14 has not been reported previously. Confirming the results of the WES, the Sanger sequencing was done in the proband and his parents. All identified pathogenic mutations were confirmed by Sanger sequencing. Two paired primers were designed as follows: F: 5´–ATCTTTGGCATCCAGTAGGC–3´; R: 5´–ACTGCAGTGGCTGAAGGTGAG–3´ (c.928G>A). F: 5´–GACCTATGATTGCGTCACTGC–3´; and R: 5´–AGAGGTGGGAGTGCGAGG–3´ (c.1864_1866dupACC). Further mutational analysis of the *NPHS1* gene of the parents showed a heterozygous mutation, c.928G>A, in the father (Fig. 2A–C) and a heterozygous mutation, c.1864_1866dupACC, in the mother (Fig. 3A–C).

**Figure 4.**
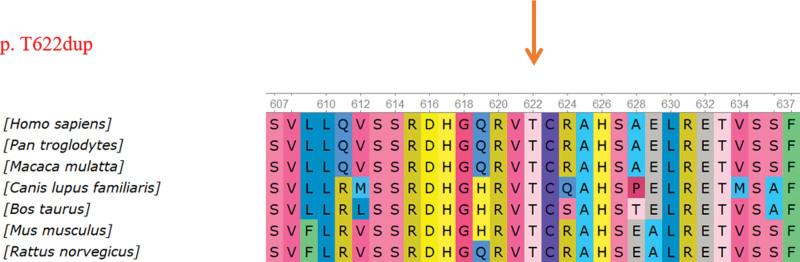
Conservative and *in silico* analysis. The protein site showing an amino acid change was found to be evolutionarily conserved across 7 species. The protein locus with the mutation is highlighted with a red arrow.

The c. 1864_1866dupACC mutation was categorized as a “Likely Pathogenic” mutation, mainly based on 4 pieces of evidence according to the ACMG guidelines.^[7]^ With regards to PM2 evidence: the frequency in the normal population database shows low-frequency variation. PM3 (Trans) evidence referred to a recessive genetic disease co-existing with another likely pathogenic variation (c.928G>A); this site was not reported in the literature/databases. Furthermore, the ClinVar database showed no pathogenicity analysis for this site. PM4 evidence referred to a change in protein length caused by the insertion of amino acid, thus disrupting protein function. PP4 evidence related to the phenotype of the patient is consistent with that of CNS. Collectively, this evidence, the circumstances of the family being analyzed, and highlighted the detection of a novel and potentially causative variant (c.1864_1866dupACC p. T622dup) in exon 14 and a previously reported mutation c.928G>in exon 8 of the *NPHS1* gene. Conservative analysis of the novel mutated amino acid sequences was carried out with the NCBI database for alignment analysis (https://www.ncbi.nlm.nih.gov/).The locus of the in-frame variant (c.1864_1866dupACC p. T622dup) was found to be evolutionarily conserved in 7 species (Fig. 4).

Gathering of relevant information, obtaining of participants consent, and protocols for this study were approved by the medical ethics committee at Guizhou provincial people hospital (approval number 2022-05). An informed written consents was obtained from parents in this subject under 18 years of age.

## 3. Discussion

In this study, an in-frame mutation (c.1864_1866dupACC) and a missense mutation (c.928G>A) in the *NPHS1* gene were detected in an infant who possessed the clinical features of CNS.

CNS is classified as either primary or secondary according to specific etiology. Primary CNS refers to glomerular lesions caused by genetic defects, often accompanied by premature delivery and a large placenta (a placenta weight >25% of the child birth weight). Secondary CNS refers to renal manifestations secondary to congenital infections (syphilis, toxoplasma gondii or cytomegalovirus) or maternal immune diseases (e.g., systemic lupus erythematosus). There are 4 types of primary CNS: CNF, diffuse mesangial sclerosis, and minimal change disease and nephrosclerosis nephropathy. The clinical description of CNF involves 2 main aspects. First, affected children are premature and have a low birth weight. Second, CNF is typically characterized by a large placenta, severe edema, abdominal distension, hypoproteinemia, massive proteinuria, and hyperlipidemia and severe infection. Most patients with CNS do not respond to any immunosuppressive therapy and treatment is largely symptomatic. More seriously, the disease progresses rapidly and develops into end-stage kidney disease within 2 to 3 years. Therefore, the early diagnosis and intervention of CNS by prenatal diagnosis are more important than treatment.

During prenatal examinations, the measurement of AFP has been used to identify congenital kidney disease since the 1970s. A large amount of proteinuria *in utero* can lead to increased AFP concentrations in the amniotic fluid and maternal serum.^[8]^ Therefore, the elevation of AFP levels and normal ultrasound images of a fetus are important for the detection and diagnosis of CNS. For example, Yan et al^[9]^ reported a fetus diagnosed with CNS by considering amniotic fluid AFP and cord blood genetic testing at 32 weeks of gestation; subsequently, the pregnant woman chose to terminate the pregnancy after genetic counseling. Another example was described by Gigante et al^[8]^ in an Italian family; their first child was diagnosed with CNF (*NPHS1* c.248 insA and p. S572N). These 2 mutations originated from the mother and father, respectively. When the molecular prenatal diagnosis was performed during the second pregnancy, the mother showed high levels of AFP in the amniotic fluid (20 MoM at 16 weeks of gestation). Subsequently, a sample of fetal DNA was analyzed for mutations; we found that the fetus was a compound heterozygote for the same mutation. This information shows that the measurement of AFP could have avoided the birth of such patients. However, it should be noted that abnormal AFP levels can also be observed in other fetal abnormalities, such as neural tube defects.^[8]^ Although AFP is not specific for CNF, it was particularly important in this case. During pregnancy, the proband mother had significantly elevated plasma levels of AFP. However, amniocentesis and prenatal diagnosis were not performed. Although AFP cannot be used as a diagnostic test for CNF, it does reflect some abnormal indicators and is therefore relevant, at least in part, and for patients with CNF.

The main mutated genes in CNS include *NPHS1, NPHS2, WTI, PLCE1*, and *LAMB2*; of these, *NPHS1* is the most common pathogenic gene.^[10]^ The *NPHS1* gene is located on chromosomal region 19q13.1 and can transcribe 4.3Kb of mRNA. Furthermore, *NPHS1* codes for the nephrin protein, and which features 1241 amino acid residues and is a key component of the interpodocyte-spanning slit diaphragm.^[1,11]^ Nephrin is a functional protein in the glomerular filtration barrier; patients who are deficient in nephrin have narrow filtration slits due to the absence or malfunction of the slit diaphragm.^[12]^ According to the literature, approximately 250 mutations have been described in the *NPHS1* gene and affect all of the domains of nephrin.^[13]^ *NPHS1* mutations are especially common in the Finnish population with an incidence as high as 1:8200, including deletions, insertions, nonsense, missense, splice site, and promoter mutations.^[2,14]^ Furthermore, mutations in this gene can lead to later-onset nephrotic syndrome and focal segmental glomerulosclerosis.^[15]^

With the development of precision medicine, whole genome sequencing is essential for accurate diagnosis. We used WES to screen a CNS proband for pathogenic factors and found a novel mutation site in the *NPHS1* gene (c.1864_1866dupACC), which forms a compound heterozygote with a missense mutation (c.928G>A). The small in-frame insertions (nonframeshift) occur in nonrepetitive sequence regions, causing amino acid duplications that alter the sequence and structure of the protein and create conformational changes that may affect protein function. In addition, the locus was evolutionarily conserved, thus suggesting that the variant was likely to be pathological. The c.928G>A (p.D310N) mutation leads to a reduced number of hydrogen bonds in the protein, thus affecting protein conformation and function. This mutation site was first reported in a Chinese family by Jie et al^[16]^ and was shown to form compound heterozygous mutations with another 2 mutations (1893–1900del 8 and G2869C). Moreover, this site has also been reported in subsequent publications. For example, Fu et al^[2]^ found a splice site mutation (IVS11 + 1G>A) and a missense mutation (c.928G>A) in the *NPHS1* gene in a Chinese child with CNS. Nguyen et al^[17]^ reported 3 heterozygous mutations (NM_0046 46: c.928G>A, c. 349 G>A, and c. 32503251 insG) in a CNS patient. Both c.1864_1866dupACC and c.928G>A are located in the Ig superfamily immunoglobulin domain. Ig superfamily domains can be divided into 4 main classes based on their structures and sequences: the variable (V), constant 1 (C1), constant 2 (C2), and intermediate (I) sets. In addition, the c. 1864_1866dupACC mutation does not feature in any known database and was categorized as “Likely Pathogenic” according to ACMG guidelines. Based on our analysis, these 2 heterozygous mutations in the *NPHS1* gene were deemed to be disease-causing mutations in this infant.

In conclusion, we report a rare case of CNS sharing uniform genotypes and similar phenotypes, thus confirming the critical role of genetic factors in CNS phenotypes. The novel mutation in this Chinese infant enriches the genetic variation and clinical phenotype of CNS and provides a basis for the molecular diagnosis, genetic counseling and prenatal diagnosis of CNS combined with genetic testing.

## Acknowledgments

We appreciate the participation of the patient and his family members in this study.

## Author contributions

**Data curation:** Dan Xie.

**Formal analysis:** Dan Xie, Jiangfen Wu.

**Investigation:** Dan Xie, Wenyi Zhang.

**Methodology:** Dan Xie, Peng Wu.

**Project administration:** Shengwen Huang, Banquan An.

**Writing – original draft:** Dan Xie, Tingting Jin.

**Writing – review & editing:** Shengwen Huang.

## References

[1] Ovunc B Ashraf S Vega-Warner V. Mutation analysis of NPHS1 in a worldwide cohort of congenital nephrotic syndrome patients. Nephron Clin Pract. 2012;120:c139–46. 22584503 10.1159/000337379 PMC5593135

[2] Fu R Gou MF Ma WH. Novel NPHS1 splice site mutations in a Chinese child with congenital nephrotic syndrome. Genet Mol Res. 2015;14:433–9. 25729976 10.4238/2015.January.23.17

[3] Jacob A Habeeb SM Herlitz L. Case report: CMV-associated congenital nephrotic syndrome. Front Pediatr. 2020;8:580178. 33330277 10.3389/fped.2020.580178 PMC7728737

[4] Hölttä T Jalanko H. Congenital nephrotic syndrome: is early aggressive treatment needed? yes. Pediatr Nephrol. 2020;35:1985–90. 32377865 10.1007/s00467-020-04578-4 PMC7501131

[5] Heeringa SF Vlangos CN Chernin G. Thirteen novel NPHS1 mutations in a large cohort of children with congenital nephrotic syndrome. Nephrol Dial Transplant. 2008;23:3527–33. 18503012 10.1093/ndt/gfn271 PMC2720813

[6] Chen Y Zhang Y Wang F. Analysis of 14 patients with congenital nephrotic syndrome. Front Pediatr. 2019;7:341. 31456999 10.3389/fped.2019.00341 PMC6700319

[7] Richards S Aziz N Bale S. Standards and guidelines for the interpretation of sequence variants: a joint consensus recommendation of the American College of Medical Genetics and Genomics and the Association for Molecular Pathology. Genet Med. 2015;17:405–24. 25741868 10.1038/gim.2015.30 PMC4544753

[8] Gigante M Greco P Defazio V. Congenital nephrotic syndrome of Finnish type: detection of new nephrin mutations and prenatal diagnosis in an Italian family. Prenat Diagn. 2005;25:407–10. 15906409 10.1002/pd.1171

[9] Yan C Qin L Liao S. Genetic variant detection and prenatal diagnosis in a Finnish family with congenital nephrotic syndrome. Zhonghua Yi Xue Yi Chuan Xue Za Zhi. 2019;36:1022–4. 31598951 10.3760/cma.j.issn.1003-9406.2019.10.018

[10] Güngör T Eroğlu FK Kargin Çakici E. Gastric duplication cyst in an infant with Finnish-type congenital nephrotic syndrome: concurrence or coincidence? Acta Clin Belg. 2021;76:155–7. 31587616 10.1080/17843286.2019.1675333

[11] Kestilä M Järvelä I. Prenatal diagnosis of congenital nephrotic syndrome (CNF, NPHS1). Prenat Diagn. 2003;23:323–4. 12673639 10.1002/pd.589

[12] Tryggvason K Patrakka J Wartiovaara J. Hereditary proteinuria syndromes and mechanisms of proteinuria. N Engl J Med. 2006;354:1387–401. 16571882 10.1056/NEJMra052131

[13] Guaragna MS Cleto TL Souza ML. NPHS1 gene mutations confirm congenital nephrotic syndrome in four Brazilian cases: a novel mutation is described. Nephrology (Carlton). 2016;21:753–7. 26560236 10.1111/nep.12667

[14] Patrakka J Kestilä M Wartiovaara J. Congenital nephrotic syndrome (NPHS1): features resulting from different mutations in Finnish patients. Kidney Int. 2000;58:972–80. 10972661 10.1046/j.1523-1755.2000.00254.x

[15] Pollak MR. Expanding the spectrum of NPHS1-associated disease. Kidney Int. 2009;76:1221–3. 19946311 10.1038/ki.2009.391

[16] Shi Y Ding J Liu J-C. [NPHS1 mutations in a Chinese family with congenital nephrotic syndrome]. Zhonghua Er Ke Za Zhi. 2005;43:805–9. 16316524

[17] Nguyen TKL Pham VD Nguyen TH. Three novel mutations in the NPHS1 gene in Vietnamese patients with congenital nephrotic syndrome. Case Rep Genet. 2017;2017:2357282. 28392951 10.1155/2017/2357282 PMC5368377

